# Models of care for eating disorders: findings from a rapid review

**DOI:** 10.1186/s40337-022-00671-1

**Published:** 2022-11-15

**Authors:** Melissa J. Pehlivan, Jane Miskovic-Wheatley, Anvi Le, Danielle Maloney, National Eating Disorders Research Consortium, Stephen Touyz, Sarah Maguire

**Affiliations:** 1 InsideOut Institute for Eating Disorders, University of Sydney, Sydney Local Health District, Sydney, Australia; 2 Healthcare Management Advisors, Melbourne, Australia

**Keywords:** Eating disorders, Patient care, Models of care, Treatment, Services, Review, Specialist, Multi-disciplinary

## Abstract

**Background:**

Delayed diagnosis, gaps in services and subsequent delays in specialist care and treatment lead to poorer health outcomes for individuals with eating disorders (EDs) and drive significant government healthcare expenditure. Given the significant disease burden associated with EDs, it is imperative that current implementation research is summarised to identify gaps in care and enable refinement for optimal patient outcomes. This review aimed to provide an updated synthesis on models of care for EDs in developed healthcare systems.

**Methods:**

This paper was conducted as part of a series of Rapid Reviews (RRs) to be published in a special series in the Journal of Eating Disorders. To provide a current and rigorous review, peer-reviewed articles published in the English language between 2009 and 2021 across three databases (ScienceDirect, PubMed and Ovid/Medline) were searched, with priority given to higher level evidence (e.g., meta-analyses, large population studies, Randomised Control Trials (RCTs)). The current review synthesises data from included studies investigating models of care for people with EDs.

**Results:**

Sixty-three studies (4.5% of the original RR) were identified, which included several diagnostic populations, the most common being Anorexia Nervosa (AN) (30.51%). Across EDs, specialist care was found to improve patient outcomes, with many patients effectively being treated in outpatient or day programs with multi-disciplinary teams, without the need for lengthy inpatient hospitalisation. Few studies investigated the interaction of different ED services (e.g., inpatient, community services, primary care), however stepped care models emerged as a promising approach to integrate ED services in a targeted and cost-effective way. Issues surrounding low treatment uptake, underdiagnosis, long waiting lists and limited hospital beds were also evident across services.

**Conclusion:**

Findings suggested further research into alternatives to traditional inpatient care is needed, with partial and shorter ‘hospitalisations’ emerging as promising avenues. Additionally, to tackle ongoing resource issues and ensure timely detection and treatment of EDs, further research into novel alternatives, such as active waiting lists or a greater role for primary care clinicians is needed.

**Plain English summary:**

This paper is part of a larger Rapid Review series carried out to guide Australia’s National Eating Disorders Research and Translation Strategy 2021–2031. Rapid reviews aim to thoroughly summarise an area of research over a short time period, typically to help with policymaking in this area. This Rapid Review summarises the evidence relating to how we care for people with eating disorders in Western healthcare systems. Topics covered include inpatient/hospital care, residential care, day programs, outpatient/community care, and referral pathways. Findings suggested specialist eating disorder services may enhance detection, referral, and patient care. Stepped care models presented as a cost-effective approach which may help with linkage between different eating disorder services. There was a trend towards shorter hospital stays and approaches which allow for greater connection with the community, such as day programs. Evidence was also found of treatment delays, due to system issues (long waiting lists, lack of accurate assessment and diagnosis) and patient-related barriers (stigma, recognition). Upskilling and involving primary care clinicians in diagnosis and referral as part of a stepped care model may help to address some of these concerns. Further efforts to improve mental health literacy and de-stigmatise help-seeking for eating disorders are needed.

**Supplementary Information:**

The online version contains supplementary material available at 10.1186/s40337-022-00671-1.

## Introduction

Eating disorders (EDs), including Anorexia Nervosa (AN) and Bulimia Nervosa (BN), are serious mental illnesses, characterised by severe disturbances in eating behaviours [1] and are particularly difficult to treat, with poor treatment outcomes for some [2], including high relapse rates [3]. Disordered eating is on the rise [4] and has been associated with increased risk of developing threshold EDs [5], and the lifetime prevalence of the latter has been reported to be 8.4% for females and 2.2% for males [6]. However, fewer than one in four of those with diagnosable EDs seek treatment [7], and those who do may live with the illness for up to 10 years for BN and 15 years for AN, before starting treatment [8]. This is in part due to a number of patient-related factors, such as illness stigma or shame, fear of losing control and poor mental health literacy [9], but also, clinician and healthcare delivery factors, such as limited ED services leading to long wait times, low engagement and drop-out [10–13]. Around half of all ED cases take a protracted course [14, 15], this proves to be an additional challenge for clinicians, who report high rates of burnout [16–18], and at a cost of the wider health system. Being a potentially chronic illness [19], EDs are particularly difficult and expensive to treat, associated with a myriad of medical complications [20, 21]. Delayed diagnosis, gaps in services and subsequent lack of availability in specialist care and treatment services all extend the duration for which the condition is untreated and lead to poorer health outcomes for the individual and significant government healthcare expenditure [22]. Further, EDs are associated with significant disease burden [23] and an elevated mortality rate among the highest of all psychiatric illnesses [24]. Hence it is vital that we evaluate the implementation of current treatments for EDs in practice to determine gaps in service delivery for refinement to optimise care and maximise cost effectiveness.

Similar to other complex conditions, those with EDs benefit from clearly defined referral pathways and comprehensive care provided by multidisciplinary teams (MDTs)[25, 26]. MDTs frequently include psychiatrists, psychologists, primary care physicians, dietitians and nurses who work together to address both the physical and psychological needs of the individual [27]. Specialist ED services are comprised by a MDT of medical and non-medical staff (e.g., psychologists) with expertise in treating EDs and the recommended treatments, who work together to set holistic treatment goals for the individual in care [12, 28–30]. Individuals with EDs may be treated in numerous settings, including primary (e.g., GPs/physicians), inpatient/hospital and outpatient/community care. Inpatient or hospital care refers to the hospitalisation (in general or specialised wards [31]) of patients to treat medical complications related to their ED [32]. Sometimes patients are also admitted without a medical emergency, rather to improve ED symptoms (e.g., break binge/purge cycle) [33], meet weight objectives for safety, or in the case of suicidal ideation [32]. When care is not received voluntarily, patients may be compelled to inpatient care under local legislation [34, 35]. Similar treatment modalities to inpatient care include residential care – where medical monitoring, meal support and therapy (individual and group) are offered in a residential setting [36] – and day programs, where patients receive supervised meal support, individual and group therapy during the day but continue residence in their homes [37]. These alternatives aim to improve patient experiences and outcomes by improving quality of life (QoL) [38] and allowing the individual to maintain connected with their social supports and day-to-day settings [37], respectively. Community or outpatient services are a step-down in intensity from day programs, typically consisting of a MDT which provides assessment and treatment, with referral to higher level care (e.g., inpatient) if needed [39]. These lower intensity alternatives (e.g., outpatient services, day programs) may allow individuals to practice their newly learned skills in their home settings, increasing generalisation of skills obtained in treatment [40].

Reviews of current service delivery for EDs have typically explored different models of care for particular ED subtypes, including primary care [in AN, BN, BED; 41], and inpatient care for AN [42] and Avoidant/Restrictive Food Intake Disorder (ARFID) [43]; as well as compulsory treatments [34], day programs [36] and the relative benefits of inpatient versus outpatient care for AN and BN [44]. Many of these reviews have relied exclusively on randomised controlled trial (RCT) data which, despite providing high quality evidence for the effectiveness of different treatments, are not without their limitations. In particular, RCTs are expensive and logistically difficult to conduct among individuals with EDs, whose urgent medical needs preclude ethical randomisation to true control conditions (e.g., waitlist, placebo) and rarity of illness, particularly for AN, complicates recruitment [45]. Further, high treatment drop-out rates in ED can lead to biased results even with best practice intent-to-treat analyses, while the highly stringent eligibility criteria of RCT investigations (e.g., no other comorbid conditions, full threshold ED diagnosis) also means that diverse or more complex ED cases are not represented in these study types [45]. Including alternate study types (e.g., cohort or case-control design) in reviews of ED models of care may provide important insights into the progression of individuals with EDs through the health system and a better representation of the types of individuals who present to care for ED.

The aim of this Rapid Review (RR) is to synthesise the literature on models of healthcare delivery for EDs across a broad range of settings (e.g., inpatient, day programs, community/outpatient) and study types (e.g., RCTs, cohort studies). This RR forms one of a series of reviews scoping the field of EDs commissioned to inform the Australian National Eating Disorders Research and Translation Strategy 2021–2031 [46]. The objective is to evaluate the current practices for ED treatment to identify gaps in current models of care and thus enable further improvement of healthcare delivery for people with EDs.

## Methods

The Australian Government Commonwealth Department of Health funded the InsideOut Institute for Eating Disorders (IOI) to develop the Australian Eating Disorders Research and Translation Strategy 2021–2031 [46] under the Psych Services for Hard-to-Reach Groups initiative (ID 4-8MSSLE). The strategy was developed in partnership with state and national stakeholders including clinicians, service providers, researchers, and experts by lived experience (including consumers and families/carers). Developed through a two-year national consultation and collaboration process, the strategy provides the roadmap to establishing EDs as a national research priority and is the first disorder-specific strategy to be developed in consultation with the Australian National Mental Health Commission. To inform the strategy, IOI commissioned Healthcare Management Advisors (HMA) to conduct a series of RRs to assess broadly, all available peer-reviewed literature on the six DSM-5 listed EDs.

A RR protocol [47] was utilised to swiftly synthesise evidence in order to guide public policy and decision-making (3). This approach has been adopted by several leading health organisations including the World Health Organisation [48] and the Canadian Agency for Drugs and Technologies in Health Rapid Response Service [49], to build a strong evidence base in a timely and accelerated manner, without compromising quality. A RR is not designed to be as comprehensive as a systematic review – it is purposive rather than exhaustive and provides actionable evidence to guide health policy [50].

The RR is a narrative synthesis and adheres to the PRISMA guidelines [51]. It is divided by topic area and presented as a series of papers. Three research databases were searched: ScienceDirect, PubMed and Ovid/Medline. To establish a broad understanding of the progress made in the field of eating disorders, and to capture the largest evidence base from the past 12 years (originally 2009–2019, but expanded to include the preceding two years), the eligibility criteria for included studies into the rapid review were kept broad. Therefore, included studies were published between 2009 and 2021, in English, and conducted within Western healthcare systems or health systems comparable to Australia in terms of structure and resourcing. The initial search and review process was conducted by three reviewers between 5 and 2019 and 16 January 2020. The re-run for the years 2020–2021 was conducted by two reviewers at the end of May 2021.

The RR had a translational research focus with the objective of identifying evidence relevant to developing optimal care pathways. Searches therefore used a Population, Intervention, Comparison, Outcome (PICO) approach to identify literature relating to population impact, prevention and early intervention, treatment, and long-term outcomes. Purposive sampling focused on high-level evidence studies such as: meta-analyses; systematic reviews; moderately sized randomised controlled studies (RCTs) (*n* > 50); moderately sized controlled-cohort studies (*n* > 50), and population-based studies (*n* > 500). However, the diagnoses ARFID and UFED necessitated less stringent eligibility criteria, due to a paucity of published articles. As these diagnoses are newly captured in the DSM-5 (released in 2013, within the allocated search timeframe), the evidence base is emerging, and fewer studies have been conducted. Thus, smaller studies (*n* = < 20) and narrative reviews of these two disorders, were also included. Grey literature, such as clinical or practice guidelines, protocol papers (without results) and Masters’ theses or dissertations, was excluded. Other sources (which may not be replicable when applying the current methodology) included the personal libraries of authors, yielding 4 additional studies (see Supplementary Materials – Additional File 1). This extra step was conducted in line with the PRISMA-S: an extension to the PRISMA Statement for Reporting Literature Searches in Systematic Reviews [52].

Full methodological details including eligibility criteria, search strategy and terms and data analysis are published in a separate protocol paper, detailing the full scope of the RR, which included a total of 1320 studies [53]. Data from included studies relating to models of care were synthesised and are presented in the current review. No further analyses were carried out on results reported here.

## Results

Sixty-three studies are included in this RR (see Supplementary Materials – Additional File 2 for a full list), 45 (71.4%) of which are primary articles. Fifty-nine of the articles related to Models of Care were identified by the original RR scoping the field of eating disorders [53], representing 4.5% of the total (1320 studies). A further four articles were identified in the write-up of this RR, by the authors. A wide range of ED diagnostic groups were covered, including AN (*n* = 21; 33.3%), BN (*n* = 7; 11.1%), ARFID (*n* = 3; 4.8%), BED (*n* = 4; 6.4%), and Night Eating Syndrome (NES) (*n* = 1; 1.6%). A number of studies included multiple ED populations (*n* = 24; 38.0%), including those with Other Specified Feeding and Eating Disorder (OSFED), and Unspecified Feeding or Eating Disorder (UFED), previously Eating Disorder Not Otherwise Specified (EDNOS) in the DSM-IV. Three studies included participants without a formal ED diagnosis (4.8%). Results are structured into the following subsections: (1) inpatient/hospital care, (2) residential care, (3) day programs, (4) outpatient/community care, and (5) referral pathways. Findings relating to full threshold EDs (e.g., AN, BN, BED, ARFID) have been group together where possible within subsections.

### Inpatient/Hospital care

Inpatient options are mostly reserved for acutely ill patients with AN and ARFID experiencing severe malnutrition, as it is considered the most practical for weight restoration in severely ill patients [42]. There was a roughly equal distribution of studies focusing on children/adolescents and adult inpatients.

The diverse presentation characteristics of people with EDs influences whether they receive inpatient treatment and for how long. Kennedy et al. (2017) found that in a patient sample of adolescents with AN and atypical AN (A-AN), those with past obesity, despite greater percentage weight loss and similar duration of illness compared to other patients, were less likely to receive hospitalisation for medical stabilisation [54]. This finding held true controlling for important covariates (e.g., age, sex, ethnicity), and mediation analyses revealed this effect occurred due to patients presenting with a higher percent median body mass index (BMI) at intake, resulting in lower odds of admission [54]. In a systematic review by Atti et al. (2021) comparing compulsorily and voluntarily treated patients, those who were compelled to inpatient treatment (i.e., under compulsory orders) had more frequent prior hospitalisations and more psychiatric comorbidities, including depression, substance abuse and self-harm [34]. However, meta-analytic evidence generated from this review indicated that despite having a lower BMI at admission and on average a 3-week longer hospital stay, compulsorily treated patients achieved similar BMI at discharge [34]. Thus, it appears even those presenting with a more complex case can improve in BMI with sufficient hospitalisation.

Individuals with severe and enduring forms of ED often require hospital care with modifications to treatment that de-emphasise weight gain (e.g., minimal weight gain to achieve medical stability) and place a greater importance on QoL and individual functioning [55]. Promisingly, a review of hospital care for those with severe and enduring EDs [defined by the authors as an illness duration of 7 years or more, with several unsuccessful treatment attempts; [56], observed improvements in patients’ core ED symptoms, weight gain and general psychopathology following hospitalisation, although the long-term benefits were inconsistent [56]. Another systematic review on hospital care of people with severe and enduring EDs concluded that there is a need for involuntary care in the case of non-acceptance among those who are particularly unwell, and that individuals with severe and enduring EDs are best treated in specialised ED wards with a MDT, as opposed to a general psychiatric ward [55]. Given the diverse needs for inpatient treatment across ED diagnoses (e.g., medical stabilisation for AN versus breaking the binge/purge cycle for BN), the subsequent section considers hospital care as it relates to the different ED diagnoses.

#### Anorexia nervosa

Evidence suggests that hospitalisation is helpful for improving weight and reducing core ED pathology, although it is unclear whether it conveys a clear advantage over less intensive care. Naab et al. reported significant improvements in ED and depressive symptoms in a large sample of adolescents and adults with AN at a specialist inpatient ED service [29]. In a retrospective cohort study of patients with AN, Meguerditchian et al. (2010) found that despite inpatients presenting with lower body mass index (BMI) and a higher number of previous suicide attempts, at 5-year follow-up, BMI and frequency of BMI normalisation were comparable between inpatients and outpatients [32]. Notably, psychological and pharmacological approaches provided in addition to behavioural weight gain during hospitalisation have not been found to improve weight gain outcomes among those with severe AN [42]. The need for inpatient care has been debated, with Herpertz-Dahlmann et al. (2014) suggesting inpatients may carry a greater risk of relapse and readmission due to the difficulties experienced by these patients in their transition back into the home after prolonged hospitalisation [40]. They found that patients who stepped down to a day program after 3-weeks of hospitalisation fared similarly to patients who continued with inpatient care until they maintained their target weight for 2 weeks, in terms of BMI at both discharge and 12-month follow-up [40]. Both groups of patients received the same evidence-based outpatient care until 12-month follow-up [40]. Similarly, in a Cochrane review, those who received shorter inpatient care followed by outpatient care reported a small but significant improvement in BMI (0.14 higher), compared with those who received solely inpatient care [44]. Longer hospitalisation stays are becoming less common among both adolescents and adults, even in countries where these services are provided due to provision of public care [32, 57].

Shorter inpatient stays may achieve similar remission (i.e., absence of symptoms) and readmission rates as that of longer inpatient stays [58, 59]. In an RCT conducted by Madden et al. (2015) comparing adolescents who received either medical stabilisation (MS) or weight restoration (WR), there were no significant differences in remission or readmission rates at discharge and 12-month follow-up [59]. The WR intervention included a longer inpatient period with the objective of getting patients to 90% of their expected body weight (EBW) prior to discharge and both groups received evidence-based outpatient care (Maudsley Family-based Therapy, FBT) for 12 months [59]. This was further supported by a secondary analysis of RCT data, which found that weight gained during hospitalisation had a minimal impact on WR in a sample of adolescents with AN at the end of outpatient treatment (9–12 months; FBT, systemic family therapy, adolescent-focused therapy) [60].

Any benefit of hospitalisation is likely to be apparent early on in hospitalisation, although there is mixed evidence as to whether this results in better longer-term outcomes post-discharge. In a secondary analysis of the Madden et al. (2015) RCT, early weight gain at 4-weeks predicted higher percent (%)EBW and remission at 12-month follow-up [61]. Similarly, Fennig et al. (2017) observed that increases in weight attained during inpatient treatment were not associated with reduced ED symptoms at discharge, including drive for thinness and weight/shape concern in a cohort of adolescents with AN, indicating a need for further outpatient/community care, targeted to address these concerns [62].

Higher calorie diets during admission may be used to safely reduce the length of hospitalisation and improve weight gain outcomes [58, 63]. Previously, it was thought that refeeding needed to commence slowly to avoid refeeding syndrome, however, two systematic reviews on refeeding in AN have suggested that higher calorie refeeding does not increase the risk of refeeding syndrome [64, 65]. In a RCT comparing higher (> 2000 kCal a day) and lower (< 1400 kCal a day) calorie refeeding in adolescents and young adults with AN or A-AN, those who received the higher calorie refeeding achieved medical stability faster (three days earlier), with greater weight gains and a shorter length of inpatient stay (an average of 8 days compared with 12 days), leading to savings of up to $19,056 USD ($25,695 AUD) per patient stay [63]. In a 1-year follow-up of this RCT [58], there were no observable differences in remission (including psychological recovery) and readmission rates, weight gain or core ED symptoms between the two groups.

#### Bulimia nervosa

Compared with AN, we found fewer publications on the treatment of BN in hospital settings [44]. Diedrich et al. (2018) showed intensive inpatient care to be effective, in terms of reducing core ED symptoms, for 75% of patients presenting with severe BN symptoms [66]. Psychotherapy-focused inpatient care was able to produce a significant reduction in ED symptoms and binge/purge symptom severity with a large effect size [66], although the study did not include a control group, precluding comparison with other service models. Further, similar to inpatient treatment for AN, length of hospitalisation for BN is not related with ED symptomology at discharge [67]. A secondary analysis study of women with BN, with an average inpatient stay of 61 days, found no association between length of inpatient duration and improved ED symptomology at discharge [67]. To note, greater symptom severity at admission in BN has been linked with longer periods of hospitalisation; however, this did not significantly improve outcomes at 18-month follow-up [67].

#### Avoidant/Restrictive food intake disorder

As in AN, hospitalisation for ARFID has a primary objective of weight and nutritional restoration. Evidence from a systematic review of inpatient care for children with ARFID indicated inpatient care was able to produce positive outcomes in terms of increased food consumption following discharge [43]. Further, findings from Kapphahn et al. (2017) indicated that patients with AFRID who were hospitalised had greater odds of weight recovery and achieved a weight corresponding to 90% of median BMI, compared with those receiving outpatient care without prior hospitalisation [68].

Generally, the required length of hospitalisation for patients with ARFID, compared to those with AN is relatively long. Stranjord et al., (2015) found that patients with ARFID required longer inpatient stays than those with AN and had a greater reliance on enteral feeding to promote WR [69, 70].Compared with children with AN, children with ARFID had significantly longer duration of illness prior to first presentation [71], despite being significantly younger. These findings were supported in two other clinical sample studies by Stranjord et al. (2015) and Forman et al. (2014) [69, 72]. However, Leiberman et al. (2019) found patients with AN and ARFID presented with similar low body weights with no significant differences between their measured %EBW, and observed an association between ARFID and previous infection involving vomiting and abdominal pain [71]. Contrarily, those with ARFID did not have more diagnosed food allergies than those with AN [71]. These findings contribute to a better understanding of ARFID, the early warning signs as well as goals and approaches to hospitalisation [71].

#### Other EDs

Evidence relating to inpatient care has been identified for AN, BN and ARFID, with little mention of other diagnoses, representing a gap in our understanding. One study identified an inpatient weight loss program incorporating psychoeducation, diet and exercise for those with NES , a type of OSFED [73]. The study found this integrated inpatient approach to be highly effective in patients with NES, with a 68% remission rate at end of inpatient treatment and rates of weight loss similar to a comparison group of obese patients without NES [73].

### Residential care

There has been relatively little evaluation of residential care for individuals with EDs [74]. For adolescent and adult AN, residential care has been found to be effective in improving BMI and core ED symptoms [74]. Notably, the biggest gains in BMI and core ED symptoms were achieved within the first 6-weeks of residential care, but these were not associated with greater post-treatment gains [74]. Instead, longer stay in residential care was associated with greater BMI change at post-treatment for adult AN [74]. A 10-year longitudinal study of residential care for AN and BN, found significant improvements in core ED symptoms, which were maintained with a mean follow-up period of 4.6 years for AN and 3.8 years for BN [75].

### Day programs

Day programs may also assist in ED recovery [37], providing patients with a sense of normalcy (maintaining connection with one’s community and home environment) and a chance to put their skills newly gained in treatment to practice in everyday settings [40]. A scoping review by Baudinet et al. (2021) observed benefits from adolescent day programs in terms of weight gain, core ED symptoms, comorbidities, psychosocial functioning, QoL and motivation to recover following treatment [37]. Follow-up data were limited but promising, with most improvements maintained 3-months later and weight gain sustained 12-months after treatment [37]. Results from a retrospective chart review of children with AN, BN and EDNOS in the UnitedStates (US) reported significant improvements in weight and core symptomology using day programs [31]. Further, Brown et al. (2018) found significant decreases in ED symptomology from baseline to 2-year follow-up in a sample of adults with severe AN and BN, who received an intervention incorporating Dialectical Behaviour Therapy (DBT), enhanced Cognitive Behaviour Therapy (CBT-E) and Acceptance and Commitment Therapy, and allowed patients to ‘step down’ into intensive outpatient care from day programs [76].

Day programs appear to be promising for individuals with AFRID. A systematic review on care for children with ARFID found day programs to produce positive outcomes in terms of increased food consumption following discharge, similar to inpatient programs [43]. An observational study of children with ARFID admitted to a day program found similar weight gain and improvements were achieved in ED psychopathology to those with AN, OSFED and UFED [70].Similarly, using a meal-based behavioural intervention combining elements of CBT, DBT and FBT, provided by a therapist-led MDT, Makhzoumi et al.’s (2019) day program obtained weight recovery in ARFID patients [77]. However, it occurred at a slower rate compared to patients with AN who also received the intervention [77]. These researchers emphasised a need to engage with gastroenterologists in the care of ARFID patients due to the significant gastrointestinal symptoms experienced by this patient cohort [77]. Further, piloting of a 5-day program for ARFID involving an MDT resulted in positive changes in meal-time behaviours and amount of food consumed in a small group of young children, compared to wait-list controls [78]. Patients with ARFID in day programs were also found to have a significantly shorter mean length of stay in the program at about 7 weeks, compared with to those with AN (approximately 12 weeks stay) [70]. Adolescent patients with AN and ARFID achieved almost identical median %BMI at discharge [70].

Evidence also suggests that day programs may be a promising alternative to inpatient treatment. A 3-year follow-up study comparing individuals with BN who received inpatient care or day program was unable to find any differences in effectiveness in terms of core ED symptoms, remission rates and general psychopathology [79]. Day programs may provide a potential cost-benefit over inpatient services [40]. Cost per day has been found to be up to 34% lower for day programs [$331 USD ($343 AUD)] compared with inpatient care [$504 USD ($523 AUD)][2014 rates; 40]. Further, unlike inpatient services, day programs allow patients to remain connected to their community and to concurrently practice their newly obtained skills in their natural settings [40].

### Outpatient/Community care

Outpatient or community care for EDs is recommended for most mild-to-moderate presentations, with the vast majority of evidence-based psychological therapies primarily provided in such settings. Implementation of an outpatient Maudsley FBT program had a substantial positive impact on patient outcomes in one Australian hospital following inpatient discharge [80]. Between 2004 and 2010, admissions for AN decreased by 56%, readmissions by 75% and total number of adolescents admitted decreased by 33% [80].

Evidence suggests that EDs can be effectively treated in the community, achieving outcomes on par with inpatient services [32, 44, 56, 72]. No significant differences were found in outcome for adolescent patients with ARFID, based on inpatient or outpatient care provided, in a retrospective chart review [72]. Further, in a review of care for individuals with severe and enduring EDs, outpatient/community services led to significant improvements in ED symptoms, which were sustained at 12-month follow-up and patients reported increased motivation to recover [56].

Generally, enteral (e.g., nasogastric feeding; NGF) feeding is administered in inpatient settings, although recently outpatient services have been piloting tube feeding interventions to treat binge/purge symptoms in individuals with BN. Enteral feeding has the objective of breaking down the binge/purge cycle by exclusively NGT followed up with gradual reintroduction of meals, allowing patients to ‘withdraw’ [81]. Daniel et al. (2014) found NGF to have benefit when delivered as part of outpatient care, with 75% of participants abstinent from binge/purge behaviours during the 3-month treatment period [81]. Further, results from an RCT providing CBT plus 2-months of NGF or CBT only to patients with BN as an outpatient service reported 81% abstinence in the combined group compared to 29% in the CBT only group [82]. Daniel et al. (2014) indicated their intervention was a novel response to a lack of access to CBT for patients with BN across France [81].

#### Multi-disciplinary & specialist care

From the preliminary evidence available, engagement of an MDT team in the care of ED patients in outpatient settings compared with psychotherapy alone is associated with more appropriate referrals and treatment completion [39]. A program implemented across 11 children’s hospitals in the US found no differences in weight recovery outcomes between patients treated with inpatient versus outpatient services under the care of an MDT [83]. In Sweden, a MDT with a family-based focus was able to achieve positive weight outcomes for most adolescent patients with AN and ENDOS using a step-down day program to outpatient treatment [84].

### Referral pathways

#### Specialist care

Evidence suggests that the availability of specialist ED services within local health care systems improves identification and care. Access to specialist care pathways in the United Kingdom (UK) led to increased detection of EDs at rates two to three times higher than in areas without a specialist ED clinic [28]. Linkages between primary care and specialist services in areas where they were available also had a significant impact on consistency and quality of care for adolescents with AN or A-AN [28]. In a Danish retrospective longitudinal two-cohort study, which examined ED service impacts on reducing mortality over a 12-year period, it was determined that the establishment of a multidisciplinary service reduced the standardised mortality ratio for AN in the region from 11.2 to 2.9 for a sample of 1,064 patients referred to the service [30].

#### Barriers to care

There are several barriers to optimal care within specialist ED services in Western countries as a result of both system and patient-related factors, such as long waiting lists for therapy due to high demand for services and stigma surrounding help seeking [22, 85, 86]. However, novel approaches such as active waiting list management, where waitlist patients are asked to opt-in by responding to a letter asking if they are still interested in seeking treatment, with non-responders being discharged, may help to reduce wait times, as one recent UK study found [87]. In their literature review, Thompson and Park (2016) identified key barriers to treatment for women, noting that certain ED populations experience different barriers to treatment. Specifically, they noted that those with AN have a greater number of patient-related barriers (e.g., cognitive rigidity, need for control) compared with those with BN and OSFED, who face greater physician (e.g., under-recognition of diagnosable EDs, lack of understanding of consequences of EDs) and social-related barriers, due to social stigma and the absence of severe and visible averse physical symptoms [88]. Some prevalence rates among clinical samples indicate patients with AN may be over-represented among treatment seeking populations in specialist settings, with over half of referrals for a state-wide ED service in South Australia for patients with AN [89, 90]. A 12-month follow-up study by Mond et al. (2009) investigated the patient-related barriers to treatment seeking among Australian women with BN, including self-recognition of an ED or eating problem. Only initial perceived functional impairment and ability to suppress emotions differentiated Australian women who self-reported receiving treatment for BN with those who did not receive treatment [91]. Specifically, those who sought treatment initially perceived a greater impairment in their functioning and ability to suppress negative emotions [91], further underscoring the importance of addressing patient-related barriers in order to increase treatment uptake.

Evidence relating to increased healthcare costs surrounding untreated EDs further underscores the importance of early identification and referral to specialist ED services. Significant healthcare utilisation and expenditure in prescription medication fills, outpatient and inpatient care for conditions associated with EDs are evident up to 7 years prior to BED diagnosis [22]. Costs peak at diagnosis, with psychiatric treatment costs for inpatient care being eight times that of healthy controls, and 16 times the costs for outpatient care of healthy controls. However, healthcare utilisation and costs decline in the years following treatment of BED and resemble that of healthy controls 4-years post-diagnosis [22]. Thus, with earlier diagnosis and treatment, improved long-term health outcomes and lower costs are likely [22].

#### Stepped care models

There is evidence supporting the value of a stepped-care model for improved outcomes and highly cost-effective ED treatment. Under the stepped-care model, patients first receive self-help, then can be “stepped up” to outpatient and then further to inpatient care if they do not respond to the preceding step [36](see Supplementary Materials – Additional File 3). A cost-utility study from Finland indicated that care delivery in alignment with a stepped-care model for individuals with BN resulted in significant improvements in health-related QoL [92]. Additionally, group psychotherapy as a second line treatment for BED after unguided self-help has been shown to reduce ED-related psychopathology, such as attachment avoidance and interpersonal problems, which are known to maintain core ED symptoms [93]. An RCT assessing the effectiveness of a stepped care model for BN in the US found stepped care to be significantly superior to usual care at 1-year follow-up in terms of binge eating and compensatory behaviours [94]. Estimated cost of stepped care delivery was $12,146 USD ($16,751 AUD) per recovered patient compared with $20,317 USD ($28,020 AUD) for intensive CBT [95]. Similarly, Herpertz-Dahlmann et al. (2014) found that treatment costs for adolescent AN patients who were stepped down to a day program after 3-weeks of inpatient care [$40,687 USD ($42,192 AUD)] were up to 20% lower, compared continued inpatient treatment [$51,629 USD ($53,539 AUD)] [2014 rates; 40]. Cost savings were evident despite stepped-care patients receiving longer care (average 16.5 weeks) compared with inpatients (average 14.6 weeks) [40].

Allen and Dalton (2011), in their systematic review of ED treatment in primary care suggest that primary care clinicians could have a greater role in service delivery as part of a stepped care model - which begins with self-help and brief intervention - if a standardised protocol for EDs was developed [96]. Bryan et al. (2021) conducted a systematic review investigating types of interventions to support adults with AN transitioning to less intensive care [97]. They observed higher drop-out rates in patients who received pharmacological treatments compared with those receiving psychological treatments (e.g., CBT, Maudsley Model of Anorexia Treatment for Adults) [97]. However, these psychological treatments only produced small non-significant improvements in weight, with inconsistent findings relating to core ED symptomology.

## Discussion

This RR synthesised the literature on models of care for EDs across a wide range of healthcare settings (e.g., inpatient, outpatient and residential care), drawing on different levels of evidence (e.g., reviews, RCTs, cohort studies) and considering multi-disciplinary care along with emerging models of care (e.g., stepped care). This RR was part of a series of reviews scoping the field of EDs [53], which identified 1324 articles. The findings related to this RR on models of care have important implications for how we care for people with EDs. The healthcare setting and type of professional involved in the care of a person with an ED contribute to clinical quality and patient outcomes.

The findings of this RR suggest the presence of specialist ED services in the system may help optimise patient outcomes [42, 98] by aiding in ED detection and referral [28, 39], and reducing mortality rates [30]. Further, evidence suggests ED patients may be effectively treated in both inpatient and outpatient/community settings with MDTs [39, 80, 83]. In particular, outpatient services are ideal for medically stable patients as they are less burdensome to the individual and family, and may allow for greater generalisation of skills learnt in therapy to everyday settings [40]. Even NGF may be successfully delivered in outpatient settings [81, 82]. Reduced hospitalisation may result in further cost savings [63] and lessen the strain on resources for inpatient care [57, 99], while improving QoL during recovery for those safe to receive adequate treatment and follow up in the community setting [32]. Although some patients may be effectively treated in primary care and non-specialist settings, it remains critical that these specialist ED services are available to support the system and provide acute care for complex cases [98], particularly for those with more rapid weight loss, such as those with past obesity, as this can infer medical risk [54].

Despite a substantial amount of literature relating to the effectiveness of outpatient settings on their own [39, 56, 72, 80–83], little information is available as to how these services should interact with primary care and inpatient services to ensure comprehensive wrap-around of care for individuals. It has been proposed that primary care clinicians play a greater role in ED service delivery [96], including in the delivery of a stepped care approach. Evidence from this review recorded symptom reductions and cost savings from stepped care models, where patients are stepped up or down depending on their responsiveness to first line treatments, such as guided self-help [92–94]. Further, emerging evidence suggests patients may safely step-down from intensive care settings (e.g., inpatient, partial hospitalisation) to outpatient care with potential cost savings [40], without compromising treatment success [44, 76, 84]. Hence, stepped care models may help ensure patients receive continuity of care whilst providing a cost-effective use of resources.

The findings also suggested a greater role for primary care clinicians in the assessment and diagnosis of EDs [88]. Greater education and training of primary care clinicians may enable them to identify and accurately refer on ED cases with less obvious physical manifestations, such as BN and OSFED [88]. However, evidence from this review suggests the burden for treatment seeking also lies with the individual [91], with significant stigma around treatment seeking for individuals with EDs playing a role [88]. Those who seek treatment tend to perceive a greater cost of illness to their functioning and emotion regulation [91], suggesting greater illness impacts and potentially insight into the negative consequences of their condition. Hence, efforts to increase the public’s mental health literacy on the consequences of EDs and normalise treatment seeking may further aid in detection and help-seeking among individuals with EDs. This is particularly important for individuals with less obvious physical ED ramifications, as treatment seeking in ED populations other than AN is quite low [89, 90].

Evidence addressing inpatient care suggests it may be used effectively to take care of especially medically unwell patients (e.g., lower BMI at admission, longer illness duration) with longer hospitalisation [32, 34, 56, 67]. However, most ED patients can be effectively treated with outpatient care [32, 44, 56, 72] or day programs [40, 43, 72, 79]. Inpatient care is generally considered necessary for WR in AN and ARFID, yet there is little difference between inpatient care and less intensive options (e.g., outpatient care, day programs) in terms of weight gain and ED symptomatology achieved following treatment [32, 42, 44, 72, 79, 83]. Indeed, length of inpatient stay has been declining [32, 57], with evidence suggesting extended hospitalisations may not be necessary [40, 44, 59, 60, 67, 74]. There is some evidence to suggest AN patients may be treated more efficiently with shorter hospitalisation stays using higher calorie diets [57, 58], as the benefits of inpatient hospitalisation generally occur early in treatment [74, 97]. Considering the incidence [57] and number of admissions for AN has been on the rise [99] since the 1990s, higher calorie refeeding may help to make more hospital beds available for additional patients and reduce healthcare expenditure costs. Although higher calorie refeeding has shown promising results in terms of weight gain and psychological recovery [58], caution is advised as NGF may have a range of unwanted psychological effects in AN, representing a lack of autonomy or signifier of illness [100].

Day programs may constitute a promising middle ground between outpatient and inpatient care, providing all the daytime support of inpatient care, but allowing the patient to stay connected to their community. Evidence from this review suggests it aids in WR [31, 37] and psychological recovery [37, 76, 79], and may result in cost benefits [40]. In ARFID, day programs may be used to reduce length of inpatient stay [69, 70]. This may be due to the increased opportunities for individuals to implement the skills and learnings from treatment in their everyday settings [40].

Evidence from this review suggests delays in treatment remain, from long waiting lists [22, 85, 86], patient-related barriers [88] and lack of accurate assessment and diagnosis in primary care [88]. At the same time, the authors of this review recognise that a key issue in this field is the high rates of burnout among clinicians of individuals with EDs [16–18]. This review has discussed some potential novel solutions to help address this shortage of available clinicians and reduce clinician workload, including the use of online self-help interventions, active waiting lists [87] or self-administered treatments like outpatient NGF [81, 82]. Further research into novel solutions is needed to help reduce treatment delays. Awareness of the negative impacts of EDs may motivate individuals to seek treatment [91, 101], and efforts to heighten public understanding of EDs may assist in treatment uptake [e.g., 102, 103]. The implementation of training initiatives or programs for primary care clinicians to identify atypical or lesser-known ED presentations may further aid in the timely detection and assessment of EDs, improving prognosis and reducing healthcare costs associated with treatment delays [22, 104, 105].

Advances within the last decade have been made in developing both evidence-based treatments [106] and leading quality care standards, such as the National Institute for Health and Care (NICE) guidelines. NICE guidelines [106] stress the importance of discussing psychological treatment options with patients and providing a referral to specialist ED services for timely assessment and treatment. Also aligned with NICE guidelines [106], this RR found timely referral to specialist ED care may optimise patient care [28, 30, 39, 42, 98]. According to NICE guidelines [106], equally important to the standard of care is how specialist services communicate and interact with each other and the patient, along with the provision of detailed care plans outlining how the different services supporting the individual will work collaboratively [106]. This RR identified gaps in the comprehensive wrap-around care for individuals with EDs, with stepped care models [92–94] and a greater role for primary care physicians [96] as potential approaches to improve ED services.

This RR has several strengths, including its’ broad breadth, covering different healthcare settings, from outpatient to inpatient services, and synthesising evidence from a range of different study designs. It aimed to provide a current review (i.e., last decade) drawing on high quality data relating to models of care which would be applicable for policy making in Western contexts. Potentially relevant articles which were (a) conducted in non-Western countries, (b) with small sample sizes (except for studies examining ARFID and OSFED), (c) using qualitative methodologies and/or published in (d) non-peer-reviewed journals, (e) prior to 2009 were not included in the methodological approach and important evidence in these studies were outside the scope. Similarly, articles not published in the English language and/or unpublished data were excluded. Further, as this RR was conducted as part of a series of RRs, the search strategy was carried out in three major databases, using broad terms (e.g., ‘treatment’) due to time constraints. Hence, lack of nuance (e.g., searching smaller, discipline-specific databases or using specific keywords such as ‘inpatient’) means some articles may have been missed. Additionally, very few included studies focused on individuals with EDs other than AN and BN, this was particularly true for the evidence relating to inpatient care. Lastly, the search strategy includes a timeline which overlaps with the use of DSM-IV and DSM-5. DSM-5 made a number of changes to ED diagnoses (e.g., removal of amenorrhea criterion in AN, lower frequency of binge episodes required in BN and BED) which meant more of those who would be classified as OSFED or UFED are given a formal ED diagnosis [107]. However, given the included studies were from 2009 onwards, the majority of included studies used the DSM-5 for diagnosis and hence is largely consistent with the current approach to ED classification.

Future research should investigate cost-effective ways to increase treatment uptake and quality of care with minimal interruption to the daily lives of individuals with EDs, such as stepped care models and day programs. It is important that care is individualised and limits the burden on the individual and their family, where removing them from work/school or their community [32] can negatively affect QoL, an important factor in ED recovery [108–112]. Further, treating patients in the community, for example, in outpatient or day programs, may help with generalisation of skills taught to manage ED thoughts and behaviours, and thus improve patient outcomes [37]. Given the high-risk medical complications [20, 21, 113]and mortality rate [24] of EDs, it is critical that ongoing research into novel treatments or service delivery frameworks and their implementation is conducted to better support this vulnerable population.

## Conclusion

This RR has identified key gaps in our care for EDs, including the integration of different healthcare services and the limited role of primary care clinicians in ED services. Promisingly, findings suggest specialist ED services can effectively treat EDs in both outpatient and day programs, with inpatient services reserved for particularly symptomatic patients. Further, for most patients, only relatively short inpatient hospitalisation is required, especially when paired with appropriate referrals to evidence-based outpatient services.

## Electronic supplementary material

Below is the link to the electronic supplementary material.

Supplementary Material 1

Supplementary Material 2

Supplementary Material 3

## Data Availability

Not applicable – all citations provided.

## List of abbreviations

A-AN: Atypical Anorexia Nervosa
AN: Anorexia Nervosa
ARFID: Avoidant Restrictive Food Intake Disorder
BED: Binge Eating Disorder
BMI: Body Mass Index
BN: Bulimia Nervosa
CBT: Cognitive Behavioural Therapy
EBW: Expected Body Weight
ED: Eating Disorder
EDNOS: Eating Disorder Not Otherwise Specified
DBT: Dialectical Behaviour Therapy
FBT: Family Based Therapy
HMA: Healthcare Management Advisors
IOI: InsideOut Institute
MDT: Multi-disciplinary Team
MS: Medical Stabilisation
NES: Night Eating Syndrome
NGF: Nasogastric Feeding
NICE: National Institute for Health and Care
OSFED: Other Specified Feeding or Eating Disorder
QoL: Quality of Life
RCT: Randomised Controlled Trial
RR: Rapid Review
UFED: Unspecified Feeding or Eating Disorder
UK: United Kingdom
US: United States
WR: Weight Restoration

## References

[1] American Psychiatric Association. Diagnostic and statistical manual of mental disorders: DSM-5. Arlington, VA2013.

[2] Steinhausen H-C. Outcome of eating disorders. In: Child and adolescent psychiatric clinics of North America [Internet]. Elsevier; 2009. [225 – 42]. 10.1016/j.chc.2008.07.013 19014869

[3] Grilo CM, Pagano ME, Stout RL, Markowitz JC, Ansell EB, Pinto A (2012). Stressful life events predict eating disorder relapse following remission: six-year prospective outcomes. Int J Eat Disord.

[4] Mannan HR, Luz FQd, Sainsbury A, Touyz SW, Hay P, editors. Escalating prevalence of comorbid obesity and binge eating: 20-year cross sectional data from South Australia from 1995 to 2015. Journal of Global Diabetes and Clinical Metabolism 2 (2): Global Conference on Obesity Treatment and Weight Management March 20–22, 2017 Las Vegas, USA; 2017.

[5] Neumark-Sztainer D, Wall M, Guo J, Story M, Haines J, Eisenberg M (2006). Obesity, disordered eating, and eating disorders in a longitudinal study of adolescents: how do dieters fare 5 years later?. J Am Diet Assoc.

[6] Galmiche M, Déchelotte P, Lambert G, Tavolacci MP (2019). Prevalence of eating disorders over the 2000–2018 period: a systematic literature review. Am J Clin Nutr.

[7] Hart LM, Granillo MT, Jorm AF, Paxton SJ (2011). Unmet need for treatment in the eating disorders: a systematic review of eating disorder specific treatment seeking among community cases. Clin Psychol Rev.

[8] Oakley-Browne MA, Elisabeth Wells J, Mcgee MA, Team NZMHSR (2006). Twelve-month and lifetime health service use in te Rau Hinengaro: the New Zealand mental health survey. Aust N Z J Psychiatry.

[9] Ali K, Farrer L, Fassnacht DB, Gulliver A, Bauer S, Griffiths KM (2017). Perceived barriers and facilitators towards help-seeking for eating disorders: a systematic review. Int J Eat Disord.

[10] Carter O, Pannekoek L, Fursland A, Allen KL, Lampard AM, Byrne SM (2012). Increased wait-list time predicts dropout from outpatient enhanced cognitive behaviour therapy (CBT-E) for eating disorders. Behav Res Ther.

[11] Byrne SM, Fursland A, Allen KL, Watson H (2011). The effectiveness of enhanced cognitive behavioural therapy for eating disorders: an open trial. Behav Res Ther.

[12] Waller G, Schmidt U, Treasure J, Murray K, Aleyna J, Emanuelli F (2009). Problems across care pathways in specialist adult eating disorder services. Psychiatr Bull.

[13] Falcón BJ, Marcoux-Louie G, Pinzon J (2019). Evaluation of the referral process and patterns to a canadian specialized eating disorders treatment program. J Can Acad Child Adolesc Psychiatry.

[14] Støving RK, Andries A, Brixen K, Bilenberg N, Hørder K (2011). Gender differences in outcome of eating disorders: a retrospective cohort study. Psychiatry Res.

[15] Eddy KT, Tabri N, Thomas JJ, Murray HB, Keshaviah A, Hastings E (2017). Recovery from anorexia nervosa and bulimia nervosa at 22-year follow-up. J Clin Psychiatry.

[16] Warren CS, Schafer KJ, Crowley ME, Olivardia R (2012). A qualitative analysis of job burnout in eating disorder treatment providers. Eat Disord.

[17] Burket RC, Schramm LL (1995). Therapists’ attitudes about treating patients with eating disorders. South Med J.

[18] Warren CS, Schafer KJ, Crowley MEJ, Olivardia R (2013). Demographic and work-related correlates of job burnout in professional eating disorder treatment providers. Psychother.

[19] Touyz S, Le Grange D, Lacey H, Hay P, Smith R, Maguire S (2013). Treating severe and enduring anorexia nervosa: a randomized controlled trial. Psychol Med.

[20] Mitchell JE (2016). Medical comorbidity and medical complications associated with binge-eating disorder. Int J Eat Disord.

[21] Westmoreland P, Krantz MJ, Mehler PS (2016). Medical complications of anorexia nervosa and bulimia. Am J Med.

[22] Watson HJ, Jangmo A, Smith T, Thornton LM, Hausswolff-Juhlin M, Norring M (2018). A register-based case-control study of health care utilization and costs in binge-eating disorder. J Psychosomat Res.

[23] Mitchison D, Morin A, Mond J, Slewa-Younan S, Hay P (2015). The bidirectional relationship between quality of life and eating disorder symptoms: a 9-year community-based study of Australian women. PLoS ONE.

[24] Arcelus J, Mitchell AJ, Wales J, Nielsen S (2011). Mortality rates in patients with anorexia nervosa and other eating disorders: a meta-analysis of 36 studies. Arch Gen Psychiatry.

[25] Suetani S, Yiu SM. M B. Defragmenting paediatric anorexia nervosa: the Flinders Medical Centre Paediatric Eating Disorder Program. Australas Psychiatry. 2015;23(3). 10.1177/1039856215579543 25838555

[26] Leite PB, Damaso AR, Poli VS, Sanches RB, Silva SGA, Fidalgo JPN (2017). Long-term interdisciplinary therapy decreases symptoms of binge eating disorder and prevalence of metabolic syndrome in adults with obesity. Nutr Res.

[27] Fogarty S, Smith CA, Hay P (2016). The role of complementary and alternative medicine in the treatment of eating disorders: a systematic review. Eat Behav.

[28] House J, Schmidt U, Craig M, Landau S, Simic M, Nicholls D (2012). Comparison of specialist and nonspecialist care pathways for adolescents with anorexia nervosa and related eating disorders. Int J Eat Disord.

[29] Naab S, Schlegl S, Korte A, Heuser J, Fumi M, Fitcher M (2013). Effectiveness of a multimodal inpatient treatment for adolescents with anorexia nervosa in comparison with adults: an analysis of a specialized inpatient setting. Eat Weight Disord.

[30] Winkler LA, Bilenberg N, Horder K, Stoving RK (2015). Does specialization of treatment influence mortality in eating disorders?--a comparison of two retrospective cohorts. Psychiatry Res.

[31] Ornstein RM, Lane-Loney SE, Hollenbeak CS (2012). Clinical outcomes of a novel, family-centred partial hospitalization program for young patients with eating disorders. Eat Weight Disord.

[32] Meguerditchian C, Samuelian-Massat C, Valéro R, Begu-Le Corroller A, Fromont I, Mancini J (2010). Inpatient treatment and anorexia nervosa outcomes. e-SPEN.

[33] Castillo M, Weiselberg E (2017). Bulimia nervosa/purging disorder. Curr Probl in Pediatr Adolesc Health Care.

[34] Atti AR, Mastellari T, Valente S, Speciani M, Panariello F, De Ronchi D (2021). Compulsory treatments in eating disorders: a systematic review and meta-analysis. Eat Weight Disord.

[35] Carney T, Tait D, Touyz S, Ingvarson M, Saunders D, Wakefield A. Managing anorexia nervosa: clinical, legal and social perspectives on involuntary treatment. Nova Science Publishers; 2006.

[36] Friedman K, Ramirez AL, Murray SB, Anderson LK, Cusack A, Boutelle KN (2016). A narrative review of outcome studies for residential and partial hospital-based treatment of eating disorders. Eur Eat Disord Rev.

[37] Baudinet J, Simic M (2021). Adolescent eating disorder day programme treatment models and outcomes: a systematic scoping review. Front Psychiatry.

[38] Delinsky S, St Germain S, Thomas J, Ellison Craigen K, Fagley W, Weigel T (2010). Naturalistic study of course, effectiveness, and predictors of outcome among female adolescents in residential treatment for eating disorders. Eat Weight Disord.

[39] Mitchell SL, Klein J, Maduramente A (2015). Assessing the impact of an eating disorders treatment team approach with college students. Eat Disord.

[40] Herpertz-Dahlmann B, Schwarte R, Krei M, Egberts K, Warnke A, Wewetzer C (2014). Day-patient treatment after short inpatient care versus continued inpatient treatment in adolescents with anorexia nervosa (ANDI): a multicentre, randomised, open-label, non-inferiority trial. Lancet.

[41] Allen S, Dalton WT (2011). Treatment of eating disorders in primary care: a systematic review. J Health Psychol.

[42] Suarez-Pinilla P, Pena-Perez C, Arbaizar-Barrenechea B, Crespo-Facorro B, Del Barrio JAG, Treasure J (2015). Inpatient treatment for anorexia nervosa: a systematic review of randomized controlled trials. J Psychiatr Pract.

[43] Sharp WG, Volkert VM, Scahill L, McCracken CE, McElhanon B. A systematic review and meta-analysis of intensive multidisciplinary intervention for pediatric feeding disorders: how standard is the standard of care? J Pediatrics. 2017;181:116 – 24.e4. 10.1016/j.jpeds.2016.10.002 27843007

[44] Hay PJ, Touyz S, Claudino AM, Lujic S, Smith CA, Madden S. Inpatient versus outpatient care, partial hospitalisation and waiting list for people with eating disorders. Cochrane Database Syst Rev. 2019(1). 10.1002/14651858.CD010827.pub2 PMC6353082 30663033

[45] Watson H, Bulik C (2013). Update on the treatment of anorexia nervosa: review of clinical trials, practice guidelines and emerging interventions. Psychol Med.

[46] InsideOut Institute for Eating Disorders. Australian eating disorders research and translation strategy 2021–2031. Sydney2021.

[47] Virginia Commonwealth University. Research guides: rapid review protocol [internet]. [cited in June 2021].

[48] Brooks SK, Webster RK, Smith LE, Woodland L, Wessely S, Greenberg N (2020). The psychological impact of quarantine and how to reduce it: rapid review of the evidence. Lancet.

[49] Canadian Agency for Drugs and Technologies in Health. About the Rapid Response Service | CADTH [cited in June 2021].

[50] Hamel C, Michaud A, Thuku M, Skidmore B, Stevens A, Nussbaumer-Streit B (2021). Defining rapid reviews: a systematic scoping review and thematic analysis of definitions and defining characteristics of rapid reviews. J Clin Epidemiol.

[51] Page MJ, Moher D, Bossuyt PM, Boutron I, Hoffmann TC, Mulrow CD, et al. PRISMA 2020 explanation and elaboration: updated guidance and exemplars for reporting systematic reviews. BMJ. 2021;372. 10.1136/bmj.n160 PMC8005925 33781993

[52] Rethlefsen ML, Kirtley S, Waffenschmidt S, Ayala AP, Moher D, Page MJ (2021). PRISMA-S: an extension to the PRISMA statement for reporting literature searches in systematic reviews. Syst Rev.

[53] Aouad P, Bryant E, Maloney D, Marks P, Le A, Russell H (2022). Informing the development of Australia’s National Eating Disorders Research and Translation Strategy: a rapid review methodology. J Eat Disorders.

[54] Kennedy GA, Forman SF, Woods ER, Hergenroeder AC, Mammel KA, Fisher MM (2017). History of overweight/obesity as predictor of care received at 1-year follow-up in adolescents with anorexia nervosa or atypical anorexia nervosa. J Adolesc Health.

[55] Hay P, Touyz S. Treatment of patients with severe and enduring eating disorders. Curr Opin Psychiatry. 2015;28(6). 10.1097/YCO.0000000000000191 26382154

[56] Kotilahti E, West M, Isomaa R, Karhunen L, Rocks T, Ruusunen A (2020). Treatment interventions for severe and enduring eating disorders: systematic review. Int J Eat Disord.

[57] Støving RK, Larsen PV, Winkler LAD, Bilenberg N, Røder ME, Steinhausen HC (2020). Time trends in treatment modes of anorexia nervosa in a nationwide cohort with free and equal access to treatment. Int J Eat Disord.

[58] Golden NH, Cheng J, Kapphahn CJ, Buckelew SM, Machen VI, Kreiter A, et al. Higher-calorie refeeding in anorexia nervosa: 1-year outcomes from a randomized controlled trial. Pediatri. 2021;147(4). 10.1542/peds.2020-037135 PMC8015147 33753542

[59] Madden S, Miskovic-Wheatley J, Wallis A, Kohn M, Lock J, Le Grange D (2015). A randomized controlled trial of in-patient treatment for anorexia nervosa in medically unstable adolescents. Psychol Med.

[60] Datta N, Matheson BE, Le Grange D, Brandt HA, Woodside B, Halmi KA, et al. Exploring differences in the role of hospitalization on weight gain based on treatment type from randomized clinical trials for adolescent anorexia nervosa. Front Psychiatry. 2020;11. 10.3389/fpsyt.2020.609675 PMC7693434 33304289

[61] Madden S, Miskovic-Wheatley J, Wallis A, Kohn M, Hay P, Touyz S (2015). Early weight gain in family‐based treatment predicts greater weight gain and remission at the end of treatment and remission at 12‐month follow‐up in adolescent anorexia nervosa. Int J Eat Disord.

[62] Fenning S, Brunstein-Klomek A, Shahar B, Sarel-Michnik Z, Hadas A (2015). Inpatient treatment has no impact on the core thoughts and perceptions in adolescents with anorexia nervosa. Early Interv Psychiatry.

[63] Garber AK, Cheng J, Accurso EC, Adams SH, Buckelew SM, Kapphahn CJ (2021). Short-term outcomes of the study of refeeding to optimize inpatient gains for patients with anorexia nervosa: a multicenter randomized clinical trial. JAMA Pediatr.

[64] Kohn MR, Madden S, Clarke SD. Refeeding in anorexia nervosa: increased safety and efficiency through understanding the pathophysiology of protein calorie malnutrition. Curr Opin Pediatr. 2011;23(4). 10.1097/MOP.0b013e3283487591 21670680

[65] Garber AK, Sawyer SM, Golden NH, Guarda AS, Katzman DK, Kohn MR (2016). A systematic review of approaches to refeeding in patients with anorexia nervosa. Int J Eat Disord.

[66] Diedrich A, Schlegl S, Greetfeld M, Fumi M, Voderholzer U (2018). Intensive inpatient treatment for bulimia nervosa: statistical and clinical significance of symptom changes. Psychother Res.

[67] Beinter I, Jacobi C. Are we overdosing treatment? Secondary findings from a study following women with bulimia nervosa after inpatient treatment. Int J Eat Disord. 2018:1–7. 10.1002/eat.22894 30070386

[68] Kapphahn CJ, Graham DA, Woods ER, Hehn R, Mammel K, Forman SF, et al. Effect of hospitalization on percent median body mass index at one year, in underweight youth with restrictive eating disorders. J Adolesc Health. 2017;61(3). 10.1016/j.jadohealth.2017.03.020 28587796

[69] Strandjord SE, Sieke EH, Richmond M, Rome ES (2015). Avoidant/restrictive food intake disorder: illness and hospital course in patients hospitalized for nutritional insufficiency. J Adolesc Health.

[70] Ornstein RM, Essayli JH, Nicely TA, Masciulli E, Lone-Lonely S. Treatment of avoidant/restrictive food intake disorder in a cohort of young patients in a partial hospitalization program for eating disorders. Int J Eat Disord. 2017:1–8. 10.1002/eat.22737 28644568

[71] Leiberman M, Houser M, Voyer A-P, Grady S, Katzman DK. Children with avoidant/restrictive food intake disorder and anorexia nervosa in a tertiary care pediatric eating disorder program: a comparative study. Int J Eat Disord. 2019:1–7. 10.1002/eat.23027 30706952

[72] Forman SF, McKenzie N, Henh R, Monge MC, Kapphahn CJ, Mammel KA (2014). Predictors of outcome at 1 year in adolescents with DSM-5 restrictive eating disorders: report of the national eating disorders quality improvement collaborative. J Adolesc Health.

[73] Dalle Grave R, Calugi S, Ruocco A, Marchesini G (2011). Night eating syndrome and weight loss outcome in obese patients. Int J Eat Disord.

[74] Hiney-Saunders K, Ousley L, Caw J, Cassinelli E, Waller G (2021). Effectiveness of treatment for adolescents and adults with anorexia nervosa in a routine residential setting. Eat Disord.

[75] Brewerton TD (2011). Long-term outcome of residential treatment for anorexia nervosa and bulimia nervosa. Eat Disord.

[76] Brown TA, Cusack A, Anderson LK, Trim J, Nakamura T, Trunko ME (2018). Efficacy of a partial hospital programme for adults with eating disorders. Eur Eat Disord Rev.

[77] Makhzoumi SH, Schreyer CC, Hansen JL, Laddaran LA, Redgrave GW, Guarda AS. Hospital course of underweight youth with ARFID treated with a meal-based behavioral protocol in an inpatient-partial hospitalization program for eating disorders. Int J Eat Disord. 2019. 10.1002/eat.23049 30779365

[78] Sharp WG, Stubbs KH, Adam H, Wells BM, Lesack RS, Criado KK (2016). Intensive, manual-based intervention for pediatric feeding disorders: results from a randomized pilot trial. J Pediatri Gastroenterol Nutr.

[79] Zeeck A, Weber S, Sandholz A, Joos A, Hartmann A (2011). Stability of long-term outcome in bulimia nervosa: a 3-year follow-up. J Clin Psychiatry.

[80] Hughes EK, Le Grange D, Court A, Yeo M, Campbell S, Whitelaw M, et al. Implementation of family-based treatment for adolescents with anorexia nervosa. J Pediatr Health Care. 2014;28(4). 10.1016/j.pedhc.2013.07.012 24055072

[81] Daniel R, Didier P, Helene P (2014). A 3-month at home tube feeding in 118 bulimia nervosa patients: a one year prospective survey in adult patients. Clin Nutr.

[82] Rigaud DJ, Brayer V, Roblot A, Brindisi M-C, Verges B (2011). Efficacy of tube feeding in binge-eating/vomiting patients: a 2-month randomized trial with 1-year follow-up. J Parenter Enteral Nutr.

[83] Forman SF, Grodin LF, Graham DA, Sylvester CJ, Rosen DS, Kappahn C (2011). An eleven site national quality improvement evaluation of adolescent medicine-based eating disorder programs: predictors of weight outcomes at one year and risk adjustment analyses. J Adolesc Health.

[84] Rosling A, Ros HS, Swenne I (2016). One-year outcome and incidence of anorexia nervosa and restrictive eating disorders among adolescent girls treated as out-patients in a family-based setting. Ups J Med Sci.

[85] Graham L, Walton M. Investigating the use of CD-rom CBT for bulimia nervosa and binge eating disorder in an NHS adult outpatient eating disorders service. Behav Cogn Psychother. 30;30(9):443–56. 10.1017/S1352465810000688 21208485

[86] Fursland A, Ecreg-Hurn DM, Byrne SM, McEvoy PM (2018). A single session assessment and psychoeducational intervention for eating disorders: impact on treatment waitlists and eating disorder symptoms. Int J Eat Disord.

[87] Jenkins PE, Turner H, Morton L (2014). Active waiting list management: potential usefulness in a community eating disorders service. Eat Disord.

[88] Thompson C (2016). Barriers to access and utilization of eating disorder treatment among women. Arch Women’s Health.

[89] Mancuso SG, Newton JR, Bosanac P, Rossell SL, Mesci JB, Castle DJ (2015). Classification of eating disorders: comparison of relative prevalence rates using DSM-IV and DSM-5 criteria. Br J Psychiatry.

[90] Wade T, Vall E, Kuek A, Altman E, Long R, Mannion (2017). Development of a new statewide eating disorder service: the role of evidence in a real world setting. Int J Eat Disord.

[91] Mond JM, Hay PJ, Darby A, Paxton SJ, Quirk F, Buttner P (2009). Women with bulimic eating disorders: when do they receive treatment for an eating problem?. J Consult Clin Psychol.

[92] Pohjolainen V, Rasanen P, Roine RP, Sintonen H, Wahbeck K, Karlsson H (2010). Cost-utility of treatment of bulimia nervosa. Int J Eat Disord.

[93] Tasca GA, Koszycki D, Brugnera A, Chyurlia L, Hammond N, Francis K, et al. Testing a stepped care model for binge-eating disorder: a two-step randomized controlled trial. Psychol Med. 2018. 10.1017/S0033291718001277 29792242

[94] Mitchell JE, Agras S, Crow S, Halmi K, Fairburn CG, Bryson S (2011). Stepped care and cognitive–behavioural therapy for bulimia nervosa: randomised trial. Br J Psychiatry.

[95] Crow SJ, Agras WS, Halmi KA, Fairburn CG, Mitchell JE, Nyman JA (2013). A cost effectiveness analysis of stepped care treatment for bulimia nervosa. Int J Eat Disord.

[96] Allen S, Dalton WT (2011). Treatment of eating disorders in primary care: A systematic review. J Health Psychol.

[97] Bryan DC, Cardi V, Willmott D, Teehan EE, Rowlands K, Treasure J (2021). A systematic review of interventions to support transitions from intensive treatment for adults with anorexia nervosa and/or their carers. Eur Eat Disord Rev.

[98] Wagner R, MacCaughelty C, Rufino K, Pack T, Poplack J, George K (2016). Effectiveness of a track-based model for treating eating disorders in a general psychiatric hospital. Bull Menn Clin.

[99] Holland J, Hall N, Yeates DGRG (2016). Trends in hospital admission rates for anorexia nervosa in Oxford (1968–2011) and England (1990–2011): database studies. J R Soc Med.

[100] Halse C, Boughtwood D, Clarke S, Honey A, Kohn M, Madden S (2005). Illuminating multiple perspectives: meanings of nasogastric feeding in anorexia nervosa. Eur Eat Disord Rev.

[101] Linardon J, Brennan L (2017). The effects of cognitive-behavioral therapy for eating disorders on quality of life: a meta-analysis. Int J Eat Disord.

[102] Gratwick-Sarll K, Bentley C (2014). Improving eating disorders mental health literacy: a preliminary evaluation of the “Should I Say Something?” workshop. Eat Disord.

[103] Bentley C, Gratwick-Sarll K, Mond J (2015). Perceived psychosocial impairment associated with eating disorder features: responses to a mental health literacy intervention. J Eat Disorders.

[104] Linville D, Aoyama T, Knoble NB, Gau J (2012). The effectiveness of a brief eating disorder training programme in medical settings. J Res Nurs.

[105] Gooding HC, Cheever E, Forman SF, Hatoun J, Jooma F, Touloumtzis C (2017). Implementation and evaluation of two educational strategies to improve screening for eating disorders in pediatric primary care. J Adolesc Health.

[106] National Guideline Alliance (2017). National institute for health and care excellence: guidelines. Eating Disorders: Recognition and Treatment.

[107] Call C, Walsh BT, Attia E. From DSM-IV to DSM-5: changes to eating disorder diagnoses. Curr Opin Pediatr. 2013;26(6). 10.1097/YCO.0b013e328365a321 24064412

[108] Nilsson K, Hägglöf B (2006). Patient perspectives of recovery in adolescent onset anorexia nervosa. Eat Disord.

[109] Jenkins J, Ogden J (2012). Becoming ‘whole’ again: a qualitative study of women’s views of recovering from anorexia nervosa. Eur Eat Disord Rev.

[110] Dawson L, Rhodes P, Touyz S (2014). Doing the impossible: the process of recovery from chronic anorexia nervosa. Qual Health Res.

[111] Federici A, Kaplan AS (2008). The patient’s account of relapse and recovery in anorexia nervosa: a qualitative study. Eur Eat Disord Rev.

[112] Patching J, Lawler J (2009). Understanding women’s experiences of developing an eating disorder and recovering: a life-history approach. Nurs Inquir.

[113] Hale MD, Logomarsino JV (2019). The use of enteral nutrition in the treatment of eating disorders: a systematic review. Eat Weight Disord.

