# Supplementary material for: Models of care for eating disorders: findings from a rapid review
Source: J Eat Disord. 2022 Nov 15;10:166. doi: 10.1186/s40337-022-00671-1 (PMC9667640; doi:10.1186/s40337-022-00671-1)
Supplement: Supplementary file 1 — Supplementary Material 1 [file 40337_2022_671_MOESM1_ESM.docx]

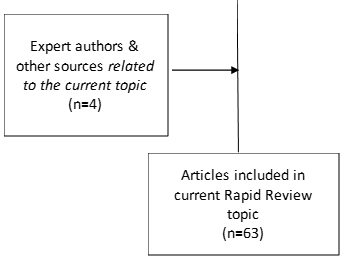


Articles identified through database searching

(n=17,757)

Articles identified through links and reference lists

(n=36)

Articles after duplicates removed

(n=9,260)

Articles screened through assessment of abstract/title

(n=9,260)

Excluded

(n=7,292)

Full text articles assessed for eligibility

(n=1,968)

Excluded

(n=660)

Expert research collaborative requested articles

(n=12)

Articles included in original Rapid Review

(n=1,320)

Identification

Screening

Eligibility

Included
