# Supplementary material for: Models of care for eating disorders: findings from a rapid review
Source: J Eat Disord. 2022 Nov 15;10:166. doi: 10.1186/s40337-022-00671-1 (PMC9667640; doi:10.1186/s40337-022-00671-1)
Supplement: Supplementary file 2 — Supplementary Material 2 [file 40337_2022_671_MOESM2_ESM.docx]

**Additional File 3**

\ General Example Stepped Care Model for Eating Disorders

Assessment

Group Therapy

Guided/Unguided Self-Help

Day Program

Inpatient Care

Individual

Therapy/ Outpatient Care

Figure shows general stepped care approach used by included studies, with patients who remain symptomatic after treatment progressing to the next step. Patients are stepped down based on clinical expertise.
