# Supplementary material for: Models of care for eating disorders: findings from a rapid review
Source: J Eat Disord. 2022 Nov 15;10:166. doi: 10.1186/s40337-022-00671-1 (PMC9667640; doi:10.1186/s40337-022-00671-1)
Supplement: Supplementary file 3 — Supplementary Material 3 [file 40337_2022_671_MOESM3_ESM.docx]

**Table 1.** Studies included in the Rapid Review

Additional File 2

**Table 1.** Studies included in the Rapid Review

| **Author, Year** | **Country** | **Aim** | **Design** | **N** | **Population** | **Outcome measure** |
| --- | --- | --- | --- | --- | --- | --- |
| Allen & Dalton, 2011 | N/A | Evaluate primary care treatments for AN, BN and BED | Review (Systematic) | 5 studies | AN, BN, BED (Mixed Cohort, All Sexes) | Core ED symptoms |
| Atti et al., 2021 | N/A | Summarise characteristics and treatment outcomes of compulsorily and voluntarily treated ED patients | Review (Systematic) | 9 studies | AN, BN, ENDOS (Mixed Cohort, All sexes) | BMI, illness duration, length of hospitalisation, mortality rates |
| Baudinet & Simic, 2021 | N/A | To review literature on adolescent ED day program treatment models and outcomes | Review (Systematic) | 49 studies | ED (Young People, All sexes) | Weight gain, core ED symptomology and related psychopathology |
| Beinter & Jacobi, 2018 | Germany | Explore the dose-response relationship in ED treatment | RCT (Secondary analysis) | 64 | BN (Adults, Females) | Core ED symptomology |
| Brewerton & Costin, 2015 | US | Examine the long-term outcome of residential treatment for ED | Longitudinal | 118 | AN; BN (Mixed Cohort, All sexes) | Weight, core ED symptoms and related psychopathology |
| Brown et al., 2018 | US | Examine the long-term outcomes of partial hospitalisation for EDs | Longitudinal | 243 | AN, BN (Adults, All sexes) | Weight, core ED symptomology and related psychopathology |
| Bryan et al., 2021 | N/A | Examine transition support interventions for stepping down from intensive care in adult AN and their carers | Review (Systematic) | 14 studies | AN (Adults, All sexes) | Dropout rates |
| Bryant-Waugh et al., 2021 | N/A | To establish an evidence-based outpatient pathway for children and young people with AFRID | Review (Narrative) | N/A | AFRID (Children, All sexes) | N/A |
| Crow et al., 2013 | US | Examine the cost-effectiveness of stepped care treatment in BN | RCT | 293 | BN (Mixed Cohort, Females) | Cost, QoL, family time burden |
| Dalle Grave et al., 2011 | Italy | Compare weight loss outcomes of obese individuals with and without NES | Observational - Case-control | 100 | NES; obese (Mixed Cohort, All sexes) | Weight loss |
| Daniel et al., 2014 | France | Examine long-term outcomes of at-home tube feeding for BN | Repeated Measure (with follow-up) | 118 | BN (Adults, Females) | Abstinence from binge/purge episodes |
| Datta et al., 2020 | US | Explore the impact of inpatient weight gain on weight outcomes in outpatient adolescent AN treatment | RCT (Secondary analysis) | 215 | AN (Adolescents, All sexes) | Weight recovery rate |
| DeBar et al., 2009 | US | Compare characteristics of participants recruited for ED trials based on recruitment strategy | Observational | 5964 | Binge episode (Adults, Females) | Clinical cut-off thresholds, BMI |
| Diedrich et al., 2018 | Germany | Examine the effectiveness of an intensive inpatient program for BN | Observational | 295 | BN (Adults, Females) | Core ED symptomology |
| Fenning et al., 2015 | Israel | Examine changes in core ED symptomology during the weight restoration phase of inpatient AN treatment | Observational | 44 | AN (Adolescents, All sexes) | Core ED symptomology |
| Forman et al., 2011 | US | Evaluate the efficacy of adolescent medicine-based ED programs for patients with restrictive EDs | Observational | 267 | AN; ARFID (Adolescents, All sexes) | Weight |
| Forman et al., 2014 | US | Examine predictors of weight restoration at 1 year follow-up after ED treatment | Observational | 700 | AN, ARFID (Adolescents, All sexes) | Weight gain |
| Garber et al., 2015 | N/A | Examine approaches to refeeding in AN | Review (Systematic) | 27 studies | AN (Mixed Cohort, All sexes) | Refeeding syndrome |
| Garber et al., 2021* | US | Examine the short-term efficacy, safety and cost of higher calorie refeeding in adolescent and adult AN | RCT | 120 | AN (Mixed Cohort, All sexes) | Medical stabilisation rate, length of stay, cost |
| Golden et al., 2021 | US | Explore the long-term outcomes of higher calorie refeeding in AN | Repeated Measure (with follow-up) | 111 | AN (Mixed Cohort, All sexes) | Remission |
| Grilo et al., 2014 | US | Examine the efficacy of delivering BED treatments in primary care settings | RCT | 104 | BED (Adults, All sexes) | Weight loss |
| Hay et al., 2015a | N/A | Assess the effectiveness of individual psychological therapies for adult AN | Systematic review/Meta-analysis | 10 studies | AN (Adults, All sexes) | Recovery |
| Hay et al., 2015b* | N/A | To summarise recent developments in the conceptualisation and treatment of individuals with severe and enduring EDs | Review (Systematic) | 28 studies | ED (Adults, All sexes) | Treatment experiences |
| Hay et al., 2019 | N/A | Assess the relative effectiveness of different care types on symptom reduction and remission rates in AN and BN | Systematic review/Meta-analysis | 5 studies | AN; BN (Mixed Cohort, All sexes) | Weight gain, treatment completion |
| Herpertz-Dahlmann et al., 2014 | Germany | Investigate the safety and efficacy of a day program following short inpatient care compared with usual inpatient care | RCT | 172 | AN (Adolescents, Females) | Weight |
| Hiney-Saunders et al., 2021 | UK | Examine the effectiveness of residential treatment for adolescent and adult AN | Observational | 98 | AN (Mixed Cohort, Females) | Weight gain |
| Holland et al., 2017 | UK | Examine long term trends in hospital admission rates for AN | Cross-sectional | N/A | AN (Mixed Cohort, All Sexes) | Hospital admission rate |
| House et al., 2015 | UK | Explore the role of specialist outpatient ED services | Observational | 287 | AN, BN, EDNOS (Adolescents, All Sexes) | Referral rates, admission, continuity of care |
| Jenkins et al., 2014 | UK | Explore effectiveness of active waiting list program for ED service | Repeated measure (without follow-up) | 108 | ED referral patients (Adults, All sexes) | Characteristics of opt-in patients, treatment attendance |
| Kapphahn et al., 2017 | US | Examine whether hospitalisation is associated with weight gain in restrictive EDs | Observational | 322 | AN, ARFID (Mixed Cohort, All sexes) | Weight gain |
| Kennedy et al., 2017 | US | Examine the link between premorbid overweight/obesity and inpatient ED care receipt | Cross-sectional | 522 | AN (Mixed Cohort, All Sexes) | Receipt of ED inpatient care |
| Kohn et al., 2011* | N/A | Summarise recent papers on refeeding in AN | Review (Narrative) | N/A | AN(Mixed Cohort, All sexes) | Refeeding syndrome |
| Kotilahti et al., 2020 | N/A | Describe the different treatment interventions and their effects in severe and enduring ED outcomes | Review (Systematic) | 23 studies | ED (Adults, All sexes) | Core ED symptom reduction |
| Leiberman et al., 2019 | Canada | Compare characteristics of children with ARFID and AN | Observational | 106 | ARFID; AN (Children, All sexes) | Illness duration, rate of admission, ED related psychopathology |
| Long et al., 2012 | UK | Compare characteristics of remitted versus non-remitted AN patients | Longitudinal | 34 | AN (Adults, All Sexes) | Core ED symptoms and related psychopathology |
| Madden et al., 2015a | Australia | Compare the effectiveness of hospitalisation for weight restoration versus medical stabilisation in AN | RCT | 82 | AN (Adolescents, All sexes) | Number of hospital days after initial admission |
| Madden et al., 2015b* | Australia | Explore whether early weight gain predicted greater weight gain and remission over time | RCT (Secondary analysis) | 82 | AN (Adolescents, All sexes) | Weight gain, remission rates |
| Makhzoumi et al., 2019 | US | Compare characteristics of youth with ARFID and AN | Observational | 275 | AN; ARFID (Adolescents, All sexes) | Initial BMI, weight gain rate, transition to partial hospitalisation |
| Meguerditchian et al., 2010 | France | Compare recovery in AN patients treated in inpatient versus those treated with outpatient care | Longitudinal | 143 | AN (Adults, Females) | Weight, recovery |
| Mitchell et al., 2011 | US | Examine whether CBT augmented with fluoxetine enhances treatment effectiveness in a stepped treatment approach | RCT | 293 | BN (Adults, All sexes) | ED symptoms and related psychopathology, psychosocial functioning |
| Mitchell et al., 2015 | US | Compare multi-disciplinary with individual care for patients with EDs | Observational | 235 | AN; BN; OSFED; UFED (Adults, All sexes) | Length of stay |
| Mond et al., 2009 | Australia | Examine the characteristics of those who do and do not receive treatment for BN-type disorders | Repeated measure (without follow-up) | 91 | BN; BED (Adults, Females) | Core ED symptomology, psychosocial functioning, mental health literacy, psych distress, defence style |
| Morris et al., 2015 | UK | Examine length of stay in specialist ED inpatient units | Observational | 206 | ED (Mixed Cohort, All sexes) | Length of stay |
| Naab et al., 2013 | Germany | Evaluate the effectiveness of a multimodal inpatient treatment in a specialised ED adult unit | Observational | 1241 | AN (Mixed Cohort, All Sexes) | Core ED symptoms, depression, weight, |
| Ornstein et al., 2012 | US | Examine the effectiveness of a partial hospitalisation program for weight gain and psychiatric symptoms in a cohort of young people with AN | Observational | 56 | AN (Young People, All sexes) | Weight gain |
| Ornstein et al., 2017 | US | Compare effectiveness of partial hospitalisation in ARFID with other EDs | Observational | 130 | AN, BN, ARFID, OSFED/UFED (Young People, All sexes) | Length of stay, BMI, core ED symptomology and related psychopathology |
| Pohjolainen et al., 2010 | Finland | Examine cost-utility of BN treatment | Repeated measure (without follow-up) | N/A | BN (Mixed Cohort, All sexes) | Cost |
| Rigaud et al., 2011 | France | Examine the efficacy of outpatient CBT plus tube-feeding versus CBT alone for adult AN and BN | RCT | 103 | AN, BN (Adults, Females) | Abstinence from binge/purge episodes |
| Rosling et al., 2016 | Sweden | Evaluate the efficacy of family-based outpatient programs for children and adolescents with restrictive EDs | Repeated Measure (with follow-up) | 168 | AN; ENDOS (Young People, Females) | Remission |
| Sharp et al., 2016 | US | Investigate the feasibility and preliminary efficacy of an intensive, manual-based behavioural feeding intervention for children with AFRID | RCT | 20 | AFRID (Children, All sexes) | Meal acceptance, meal disruptions, grams consumed |
| Sharp et al., 2017 | N/A | Explore models of care for paediatric feeding disorders | Systematic review/Meta-analysis | 11 studies | ARFID (Young People, All sexes) | Food intake, growth status, feeding behaviour |
| Stoving et al., 2020 | Denmark | Explore time-trends in treatment modes of AN patients | Longitudinal | 7505 | AN (Mixed Cohort, All sexes) | Number of inpatient days, number of admissions |
| Strandjord et al., 2015 | US | Examine remission rates of patients hospitalised for restrictive EDs | Observational | 41 | ARFID; AN (Mixed Cohort, All sexes) | Remission rate |
| Suarez-Pinilla et al., 2015 | N/A | Review RCTs of add-on inpatient treatment programs | Review (Systematic) | 18 studies | AN (Mixed Cohort, All Sexes) | Weight gain |
| Suetani et al., 2015 | Australia | Describe the establishment and main characteristics of a new paediatric ED program for child and adolescent AN | Model of Care | N/A | AN (Young People, All Sexes) |  |
| Tasca et al., 2018 | Canada | Examine the efficacy of a two-stepped care approach for BED | RCT | 135 | BED (Mixed Cohort, All sexes) | Core ED symptoms and related psychopathology |
| Thompson & Park, 2014 | N/A | Examine treatment barriers for women with AN, BN, OSFED | Review (Narrative) | N/A | AN, BN, OSFED (Adults, Females) | Treatment barriers |
| Wade et al., 2017 | Australia | Investigate the functioning of a newly developed state-wide service for EDs | Observational | 292 | AN; BN; OSFED (Mixed Cohort, All sexes) | Discharge rate; ED diagnosis |
| Wagner et al., 2016 | US | Examine the effectiveness of a track-based model for treating EDs at a general inpatient psychiatric hospital | Repeated measure (without follow-up) | 176 | ED referral patients (Adults, All sexes) | ED risk |
| Watson et al., 2016 | Sweden | Compare prescription medication utilization in those with and without BED | Observational - Case-control | 2618 | BED (Mixed Cohort, All sexes) | Prescriptions, prescription fill |
| Watson et al., 2018 | Sweden | Compare healthcare utilization and expenditure in people with and without BED. | Observational - Case-control | 3509 | BED (Mixed Cohort, All sexes) | Healthcare expenditure |
| Winkler et al., 2015 | Denmark | Compare mortality rates in sample of ED patients treated with and without MDT care | Observational - Cohort study | 998 | ED (Mixed Cohort, All sexes) | Mortality rates |
| Zeeck et al., 2011 | Germany | Examine long-term outcomes of BN patients treated in inpatient or partial hospitalisation programs | Longitudinal | 43 | BN (Adults, All sexes) | Core ED symptomology and related psychopathology, remission rates |

*Notes.**indicates articles found in the write-up of this review.
